# 4-Benzyl-3-(thio­phen-2-yl)-4,5-di­hydro-1*H*-1,2,4-triazole-5-thione

**DOI:** 10.1107/S1600536813009501

**Published:** 2013-04-13

**Authors:** Mona M. Al-Shehri, Ali A. El-Emam, Nasser R. El-Brollosy, Seik Weng Ng, Edward R. T. Tiekink

**Affiliations:** a Department of Pharmaceutical Chemistry, College of Pharmacy, King Saud University, Riyadh 11451, Saudi Arabia; b Department of Chemistry, University of Malaya, 50603 Kuala Lumpur, Malaysia; c Chemistry Department, Faculty of Science, King Abdulaziz University, PO Box 80203 Jeddah, Saudi Arabia

## Abstract

In the title compound, C_13_H_11_N_3_S_2_, the triazole and thio­phene rings are coplanar [dihedral angle = 6.22 (13)°]. By contrast, the phenyl ring is perpendicular to the triazole ring [dihedral angle = 85.58 (13)°], so that the mol­ecule has an L-shape. The thio­phene S atom is *syn* with the ring imine N atom. In the crystal, eight-membered {⋯HNCS}_2_ synthons form between centrosymmetrically related mol­ecules, leading to dimeric aggregates that are connected into a supra­molecular layer parallel to (101) by π–π inter­actions between centrosymmetrically related triazole rings [centroid–centroid distance = 3.6091 (15) Å] and C—H⋯π inter­actions.

## Related literature

For the pharmacological properties (anti-inflammatory, anti-microbial and anti-cancer) of 1,2,4-triazole derivatives, see: El-Emam & Ibrahim (1991 ▶); Navidpour *et al.* (2006 ▶); Kumar *et al.* (2010 ▶); Wang *et al.* (2011 ▶). For a related structure, see: Zareef *et al.* (2008 ▶).
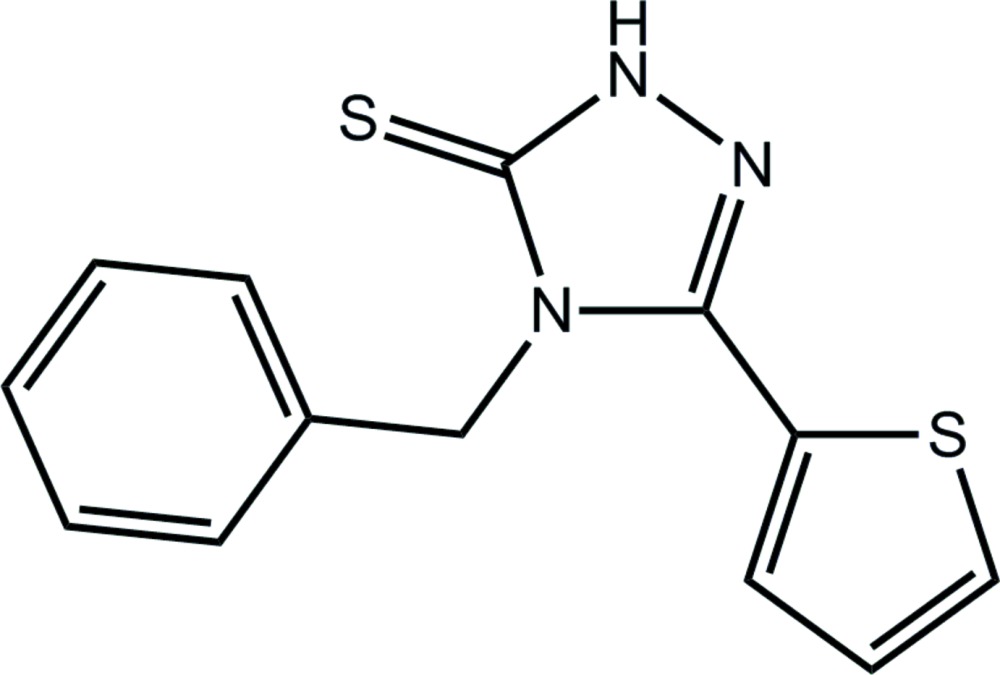


## Experimental

### 

#### Crystal data

C_13_H_11_N_3_S_2_
*M* *_r_* = 273.37
Monoclinic, 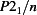

*a* = 13.422 (2) Å
*b* = 6.1670 (7) Å
*c* = 16.596 (2) Å
β = 111.972 (15)°
*V* = 1273.9 (3) Å^3^
*Z* = 4
Mo *K*α radiation
μ = 0.40 mm^−1^
*T* = 295 K
0.30 × 0.05 × 0.05 mm

#### Data collection

Agilent SuperNova Dual diffractometer with an Atlas detector
Absorption correction: multi-scan (*CrysAlis PRO*; Agilent, 2011 ▶) *T*_min_ = 0.806, *T*_max_ = 1.000
6460 measured reflections
2937 independent reflections
2088 reflections with *I* > 2σ(*I*)
*R*_int_ = 0.036

#### Refinement

*R*[*F* ^2^ > 2σ(*F* ^2^)] = 0.045
*wR*(*F* ^2^) = 0.109
*S* = 1.02
2937 reflections
167 parameters
1 restraint
H atoms treated by a mixture of independent and constrained refinement
Δρ_max_ = 0.24 e Å^−3^
Δρ_min_ = −0.27 e Å^−3^

### 

Data collection: *CrysAlis PRO* (Agilent, 2011 ▶); cell refinement: *CrysAlis PRO*; data reduction: *CrysAlis PRO*; program(s) used to solve structure: *SHELXS97* (Sheldrick, 2008 ▶); program(s) used to refine structure: *SHELXL97* (Sheldrick, 2008 ▶); molecular graphics: *ORTEP-3 for Windows* (Farrugia, 2012 ▶) and *DIAMOND* (Brandenburg, 2006 ▶); software used to prepare material for publication: *publCIF* (Westrip, 2010 ▶).

## Supplementary Material

Crystal structure: contains datablock(s) global, I. DOI: 10.1107/S1600536813009501/hg5308sup1.cif

Structure factors: contains datablock(s) I. DOI: 10.1107/S1600536813009501/hg5308Isup2.hkl

Supplementary material file. DOI: 10.1107/S1600536813009501/hg5308Isup3.cml

Additional supplementary materials:  crystallographic information; 3D view; checkCIF report

## Figures and Tables

**Table 1:** Hydrogen-bond geometry (Å, °) *Cg*1 is the centroid of the C8–C13 ring.

*D*—H⋯*A*	*D*—H	H⋯*A*	*D*⋯*A*	*D*—H⋯*A*
N2—H2⋯S2^i^	0.88 (1)	2.43 (1)	3.297 (2)	169 (2)
C13—H13⋯*Cg*1^ii^	0.93	2.94	3.636 (3)	133

Symmetry codes: (i) 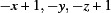; (ii) 
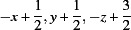
.

**Fig. 1.**
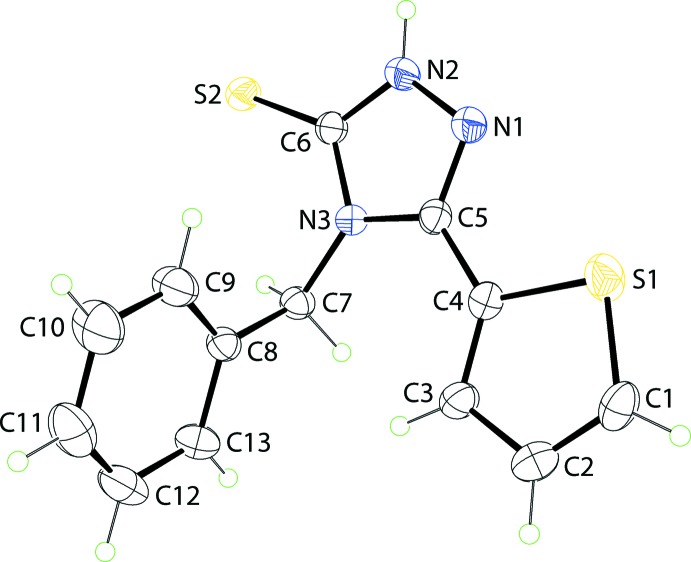
The molecular structure of (I) showing the atom-labelling scheme and displacement ellipsoids at the 35% probability level.

**Fig. 2.**
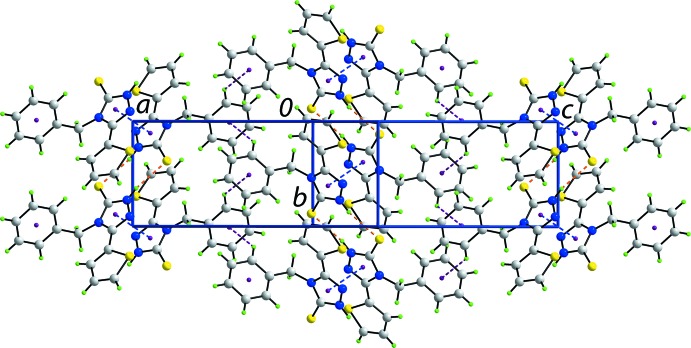
A view of the supramolecular layer in (I), which is sustained by N—H···S hydrogen bonds as well as by π—π and C—H···π interactions shown as orange, blue and purple dashed lines, respectively.

**Fig. 3.**
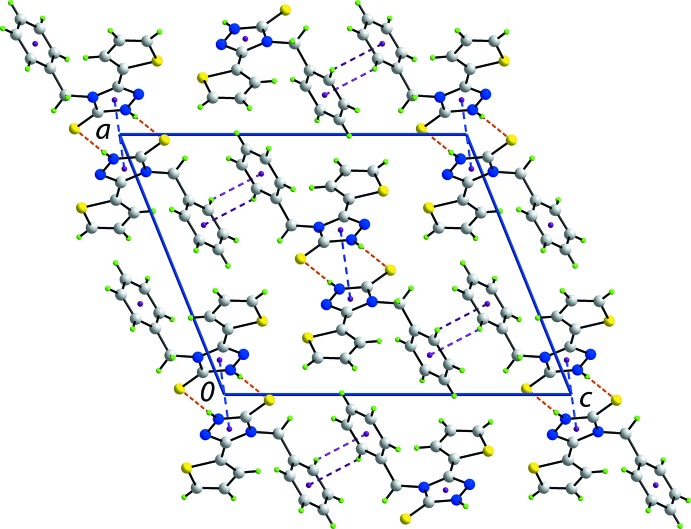
View of the unit-cell contents in projection down the b axis of (I), highlighting the stacking of layers. The N—H···S hydrogen bonds as well as by π—π and C—H···π interactions shown as orange, blue and purple dashed lines, respectively.

## supplementary crystallographic information

### Comment

In continuation of research into the chemical and pharmacological properties of 1,2,4-triazole derivatives (El-Emam & Ibrahim, 1991; Navidpour *et al.*, 2006; Kumar *et al.*, 2010; Wang *et al.*, 2011), we describe herein the X-ray crystal structure determination of the title compound, (I).

In (I), Fig. 1, the triazole ring is plane (r.m.s. deviation = 0.008 Å) and the thione-S2 atom lies 0.030 (1) Å out of the plane. The thiophene ring is co-planar with the triazole ring [dihedral angle = 6.22 (13)°] and the latter forms a dihedral of 85.58 (13)° with the phenyl ring. The thiophene-S1 atom is *syn* with the ring imine-N2 atom. Overall, the molecule has the shape of the letter *L*. A similar conformation was found in the analogous furanyl compound for which two molecules comprise the asymmetric unit and which was characterized as an hydrate (Zareef *et al.*, 2008).

In the crystal packing, centrosymmetrically related molecules aggregate into dimers *via* N—H···S hydrogen bonds that lead to eight-membered {···HNCS}_2_ synthons, Table 1. The dimers are connected into rows along the *b* axis by π—π interactions between centrosymmetrically related triazole rings [inter-centroid distance = 3.6091 (15) Å for symmetry operation: 1 - *x*, 1 - *y*, 1 - *z*]. Projecting out on either side of the row are the phenyl groups that inter-digitate with translationally related rows to enable the formation of edge-to-face C—H···π interactions, Table 1, that result in a supramolecular layer parallel to (1 0 1), Fig. 2. Layers stack with no specific interactions between them, Fig. 3.

### Experimental

A mixture of thiophene-2-carbohydrazide (1.42 g, 0.01 mol), benzyl isothiocyanate (1.49 g, 0.01 mol), in ethanol (10 ml) was heated under reflux with stirring for 1 h after which the solvent was distilled off *in vacuo*. Aqueous sodium hydroxide solution (10%, 15 ml) was added to the residue and the mixture was heated under reflux for 2 h then filtered hot. On cooling, the mixture was acidified with hydrochloric acid and the precipitated crude product was filtered, washed with water, dried and crystallized from aqueous ethanol to yield 2.32 g (85%) of the title compound as colourless crystals. *M*.pt: 515–517 K. Single crystals suitable for X-ray analysis were obtained by slow evaporation of its CHCl_3_:EtOH (1:1; 10 ml) solution at room temperature. ^1^H NMR (DMSO-d_6_, 500.13 MHz): δ 5.51 (s, 2H, CH_2_), 7.13–7.14 (m, 3H, Ar—H), 7.27–7.40 (m, 4H, Ar—H & thiophene-H), 7.77 (d, 1H, thiophene-H, J = 4.0 Hz), 14.21 (s, 1H, SH, thiol tautomer). ^13^C NMR (DMSO-d_6_, 125.76 MHz): δ 46.74 (CH_2_), 126.15, 126.33, 127.51, 128.19, 128.72, 128.86, 129.90, 135.40 (Ar—C & thiophene-C), 146.31 (triazole C-3), 168.31 (triazole C-5).

### Refinement

The C-bound H-atoms were placed in calculated positions [C—H = 0.93 to 0.97 Å, *U*_iso_(H) = 1.2*U*_eq_(C)] and were included in the refinement in the riding model approximation. The N-bound H-atom was refined with the distance restraint N—H = 0.88±0.01 Å.

### Crystal data

**Table:**

C_13_H_11_N_3_S_2_	*F*(000) = 568
*M**_r_* = 273.37	*D*_x_ = 1.425 Mg m^−^^3^
Monoclinic, *P*2_1_/*n*	Mo *K*α radiation, λ = 0.71073 Å
Hall symbol: -P 2yn	Cell parameters from 1615 reflections
*a* = 13.422 (2) Å	θ = 3.0–27.5°
*b* = 6.1670 (7) Å	µ = 0.40 mm^−^^1^
*c* = 16.596 (2) Å	*T* = 295 K
β = 111.972 (15)°	Prism, colourless
*V* = 1273.9 (3) Å^3^	0.30 × 0.05 × 0.05 mm
*Z* = 4	

### Data collection

**Table:**

Agilent SuperNova Dual diffractometer with an Atlas detector	2937 independent reflections
Radiation source: SuperNova (Mo) X-ray Source	2088 reflections with *I* > 2σ(*I*)
Mirror monochromator	*R*_int_ = 0.036
Detector resolution: 10.4041 pixels mm^-1^	θ_max_ = 27.6°, θ_min_ = 3.3°
ω scan	*h* = −12→17
Absorption correction: multi-scan (*CrysAlis PRO*; Agilent, 2011)	*k* = −8→5
*T*_min_ = 0.806, *T*_max_ = 1.000	*l* = −21→19
6460 measured reflections	

### Refinement

**Table:**

Refinement on *F*^2^	Primary atom site location: structure-invariant direct methods
Least-squares matrix: full	Secondary atom site location: difference Fourier map
*R*[*F*^2^ > 2σ(*F*^2^)] = 0.045	Hydrogen site location: inferred from neighbouring sites
*wR*(*F*^2^) = 0.109	H atoms treated by a mixture of independent and constrained refinement
*S* = 1.02	*w* = 1/[σ^2^(*F*_o_^2^) + (0.0415*P*)^2^ + 0.2539*P*] where *P* = (*F*_o_^2^ + 2*F*_c_^2^)/3
2937 reflections	(Δ/σ)_max_ < 0.001
167 parameters	Δρ_max_ = 0.24 e Å^−^^3^
1 restraint	Δρ_min_ = −0.27 e Å^−^^3^

### Special details

**Table:**

Geometry. All e.s.d.'s (except the e.s.d. in the dihedral angle between two l.s. planes) are estimated using the full covariance matrix. The cell e.s.d.'s are taken into account individually in the estimation of e.s.d.'s in distances, angles and torsion angles; correlations between e.s.d.'s in cell parameters are only used when they are defined by crystal symmetry. An approximate (isotropic) treatment of cell e.s.d.'s is used for estimating e.s.d.'s involving l.s. planes.
Refinement. Refinement of *F*^2^ against ALL reflections. The weighted *R*-factor *wR* and goodness of fit *S* are based on *F*^2^, conventional *R*-factors *R* are based on *F*, with *F* set to zero for negative *F*^2^. The threshold expression of *F*^2^ > σ(*F*^2^) is used only for calculating *R*-factors(gt) *etc*. and is not relevant to the choice of reflections for refinement. *R*-factors based on *F*^2^ are statistically about twice as large as those based on *F*, and *R*- factors based on ALL data will be even larger.

### Fractional atomic coordinates and isotropic or equivalent isotropic displacement parameters (Å^2^)

**Table:**

	*x*	*y*	*z*	*U*_iso_*/*U*_eq_	
S1	0.21756 (5)	0.78528 (13)	0.30574 (4)	0.0548 (2)	
S2	0.47823 (5)	0.12218 (10)	0.62298 (3)	0.03891 (18)	
N1	0.34574 (15)	0.4060 (3)	0.39839 (11)	0.0395 (5)	
N2	0.40554 (15)	0.2515 (3)	0.45408 (12)	0.0383 (5)	
H2	0.4302 (18)	0.138 (3)	0.4349 (15)	0.052 (8)*	
N3	0.35676 (13)	0.4651 (3)	0.53331 (10)	0.0304 (4)	
C1	0.14130 (18)	0.9997 (4)	0.31144 (16)	0.0475 (6)	
H1	0.1054	1.0904	0.2648	0.057*	
C2	0.1387 (2)	1.0233 (4)	0.39097 (16)	0.0492 (6)	
H2A	0.1008	1.1330	0.4054	0.059*	
C3	0.19944 (19)	0.8647 (4)	0.45051 (14)	0.0426 (6)	
H3	0.2054	0.8574	0.5081	0.051*	
C4	0.24840 (16)	0.7233 (4)	0.41373 (13)	0.0346 (5)	
C5	0.31583 (17)	0.5357 (4)	0.44809 (13)	0.0323 (5)	
C6	0.41379 (16)	0.2783 (3)	0.53621 (13)	0.0315 (5)	
C7	0.34971 (17)	0.5686 (4)	0.61041 (13)	0.0344 (5)	
H7A	0.3586	0.7238	0.6065	0.041*	
H7B	0.4085	0.5168	0.6614	0.041*	
C8	0.24552 (17)	0.5271 (4)	0.62246 (12)	0.0344 (5)	
C9	0.1871 (2)	0.3419 (5)	0.59316 (17)	0.0534 (7)	
H9	0.2110	0.2391	0.5635	0.064*	
C10	0.0931 (2)	0.3049 (5)	0.6069 (2)	0.0708 (9)	
H10	0.0540	0.1787	0.5863	0.085*	
C11	0.0579 (2)	0.4550 (6)	0.65120 (19)	0.0665 (8)	
H11	−0.0056	0.4316	0.6602	0.080*	
C12	0.1160 (2)	0.6383 (5)	0.68195 (17)	0.0595 (8)	
H12	0.0928	0.7387	0.7129	0.071*	
C13	0.2092 (2)	0.6763 (4)	0.66753 (15)	0.0474 (6)	
H13	0.2479	0.8029	0.6882	0.057*	

### Atomic displacement parameters (Å^2^)

**Table:**

	*U*^11^	*U*^22^	*U*^33^	*U*^12^	*U*^13^	*U*^23^
S1	0.0569 (4)	0.0694 (5)	0.0403 (3)	0.0206 (4)	0.0206 (3)	0.0166 (3)
S2	0.0448 (3)	0.0349 (3)	0.0365 (3)	0.0064 (3)	0.0146 (2)	0.0030 (3)
N1	0.0467 (11)	0.0384 (11)	0.0365 (10)	0.0075 (9)	0.0190 (8)	0.0045 (9)
N2	0.0453 (11)	0.0369 (11)	0.0369 (10)	0.0088 (9)	0.0204 (9)	0.0021 (9)
N3	0.0335 (9)	0.0279 (9)	0.0318 (9)	0.0017 (8)	0.0145 (7)	−0.0002 (8)
C1	0.0385 (13)	0.0476 (15)	0.0501 (14)	0.0047 (12)	0.0095 (10)	0.0177 (12)
C2	0.0478 (14)	0.0404 (14)	0.0561 (15)	0.0098 (12)	0.0155 (12)	0.0040 (12)
C3	0.0500 (14)	0.0378 (13)	0.0386 (12)	0.0104 (12)	0.0148 (10)	0.0046 (11)
C4	0.0328 (11)	0.0346 (12)	0.0355 (11)	−0.0007 (10)	0.0117 (9)	0.0036 (10)
C5	0.0345 (11)	0.0308 (11)	0.0313 (10)	−0.0024 (10)	0.0120 (9)	0.0009 (10)
C6	0.0309 (11)	0.0301 (11)	0.0355 (11)	−0.0029 (9)	0.0148 (9)	−0.0015 (9)
C7	0.0371 (12)	0.0337 (12)	0.0314 (10)	0.0001 (10)	0.0116 (9)	−0.0031 (9)
C8	0.0371 (12)	0.0363 (12)	0.0307 (10)	0.0035 (10)	0.0137 (9)	0.0008 (10)
C9	0.0547 (16)	0.0503 (16)	0.0651 (16)	−0.0097 (13)	0.0339 (13)	−0.0164 (13)
C10	0.0595 (18)	0.073 (2)	0.092 (2)	−0.0239 (17)	0.0418 (17)	−0.0182 (19)
C11	0.0493 (16)	0.089 (2)	0.0722 (18)	0.0006 (17)	0.0350 (14)	0.0030 (18)
C12	0.0588 (17)	0.073 (2)	0.0550 (15)	0.0159 (16)	0.0313 (13)	−0.0069 (15)
C13	0.0531 (15)	0.0455 (15)	0.0458 (13)	0.0023 (12)	0.0211 (11)	−0.0120 (12)

### Geometric parameters (Å, º)

**Table:**

S1—C1	1.696 (3)	C4—C5	1.448 (3)
S1—C4	1.725 (2)	C7—C8	1.507 (3)
S2—C6	1.678 (2)	C7—H7A	0.9700
N1—C5	1.315 (3)	C7—H7B	0.9700
N1—N2	1.361 (3)	C8—C9	1.368 (3)
N2—C6	1.336 (3)	C8—C13	1.384 (3)
N2—H2	0.881 (10)	C9—C10	1.382 (4)
N3—C6	1.374 (3)	C9—H9	0.9300
N3—C5	1.382 (2)	C10—C11	1.372 (4)
N3—C7	1.464 (2)	C10—H10	0.9300
C1—C2	1.341 (3)	C11—C12	1.359 (4)
C1—H1	0.9300	C11—H11	0.9300
C2—C3	1.412 (3)	C12—C13	1.379 (4)
C2—H2A	0.9300	C12—H12	0.9300
C3—C4	1.366 (3)	C13—H13	0.9300
C3—H3	0.9300		
			
C1—S1—C4	91.71 (11)	N3—C6—S2	127.78 (15)
C5—N1—N2	103.94 (17)	N3—C7—C8	114.11 (17)
C6—N2—N1	114.13 (18)	N3—C7—H7A	108.7
C6—N2—H2	124.6 (16)	C8—C7—H7A	108.7
N1—N2—H2	121.0 (16)	N3—C7—H7B	108.7
C6—N3—C5	107.63 (16)	C8—C7—H7B	108.7
C6—N3—C7	123.51 (17)	H7A—C7—H7B	107.6
C5—N3—C7	128.77 (18)	C9—C8—C13	118.4 (2)
C2—C1—S1	112.20 (18)	C9—C8—C7	122.1 (2)
C2—C1—H1	123.9	C13—C8—C7	119.4 (2)
S1—C1—H1	123.9	C8—C9—C10	121.1 (3)
C1—C2—C3	113.1 (2)	C8—C9—H9	119.5
C1—C2—H2A	123.4	C10—C9—H9	119.5
C3—C2—H2A	123.4	C11—C10—C9	119.7 (3)
C4—C3—C2	112.2 (2)	C11—C10—H10	120.1
C4—C3—H3	123.9	C9—C10—H10	120.1
C2—C3—H3	123.9	C12—C11—C10	119.9 (3)
C3—C4—C5	131.97 (19)	C12—C11—H11	120.1
C3—C4—S1	110.76 (16)	C10—C11—H11	120.1
C5—C4—S1	117.24 (16)	C11—C12—C13	120.4 (3)
N1—C5—N3	110.66 (19)	C11—C12—H12	119.8
N1—C5—C4	122.11 (18)	C13—C12—H12	119.8
N3—C5—C4	127.23 (19)	C12—C13—C8	120.4 (3)
N2—C6—N3	103.62 (17)	C12—C13—H13	119.8
N2—C6—S2	128.59 (17)	C8—C13—H13	119.8
			
C5—N1—N2—C6	0.3 (3)	N1—N2—C6—N3	−1.0 (2)
C4—S1—C1—C2	−0.2 (2)	N1—N2—C6—S2	178.98 (16)
S1—C1—C2—C3	−0.2 (3)	C5—N3—C6—N2	1.3 (2)
C1—C2—C3—C4	0.7 (3)	C7—N3—C6—N2	−175.41 (18)
C2—C3—C4—C5	−178.7 (2)	C5—N3—C6—S2	−178.73 (16)
C2—C3—C4—S1	−0.8 (3)	C7—N3—C6—S2	4.6 (3)
C1—S1—C4—C3	0.61 (19)	C6—N3—C7—C8	−102.1 (2)
C1—S1—C4—C5	178.87 (18)	C5—N3—C7—C8	81.9 (3)
N2—N1—C5—N3	0.5 (2)	N3—C7—C8—C9	29.0 (3)
N2—N1—C5—C4	−179.08 (19)	N3—C7—C8—C13	−153.6 (2)
C6—N3—C5—N1	−1.2 (2)	C13—C8—C9—C10	0.9 (4)
C7—N3—C5—N1	175.28 (19)	C7—C8—C9—C10	178.3 (2)
C6—N3—C5—C4	178.4 (2)	C8—C9—C10—C11	−0.5 (5)
C7—N3—C5—C4	−5.1 (3)	C9—C10—C11—C12	−0.6 (5)
C3—C4—C5—N1	172.4 (2)	C10—C11—C12—C13	1.2 (5)
S1—C4—C5—N1	−5.4 (3)	C11—C12—C13—C8	−0.8 (4)
C3—C4—C5—N3	−7.2 (4)	C9—C8—C13—C12	−0.3 (3)
S1—C4—C5—N3	175.03 (17)	C7—C8—C13—C12	−177.8 (2)

### Hydrogen-bond geometry (Å, º)

Cg1 is the centroid of the C8–C13 ring.

**Table:**

*D*—H···*A*	*D*—H	H···*A*	*D*···*A*	*D*—H···*A*
N2—H2···S2^i^	0.88 (1)	2.43 (1)	3.297 (2)	169 (2)
C13—H13···*Cg*1^ii^	0.93	2.94	3.636 (3)	133

Symmetry codes: (i) −*x*+1, −*y*, −*z*+1; (ii) −*x*+1/2, *y*+1/2, −*z*+3/2.
